# A Two-Stage Localization Scheme with Partition Handling for Data Tagging in Underwater Acoustic Sensor Networks

**DOI:** 10.3390/s19092135

**Published:** 2019-05-08

**Authors:** Tariq Islam, Yong Kyu Lee

**Affiliations:** Department of Computer Science and Engineering, Dongguk University-Seoul, Seoul 04620, Korea; tariqislam20@yahoo.com

**Keywords:** underwater acoustic sensor networks, localization, mobility, GPS, data-tagging

## Abstract

Knowledge about the geographic coordinates of underwater sensor nodes is of primary importance for many applications and protocols of under water sensor networks (UWSNs) thus making localization of sensor nodes a crucial part of underwater network design. In case of mobile underwater sensor network, location estimation becomes challenging not only due to the need for periodic tracking of nodes, but also due to network partitioning caused by the pseudo-random mobility of nodes. Our proposed technique accomplishes the task of localization in two stages: (1) relative localization of sensor nodes with respect to a reference node at regular intervals during sensing operation. (2) Offline absolute localization of sensor nodes using absolute coordinates of the reference node and relative locations estimated during stage 1. As our protocol deals with mobile underwater sensor networks that may introduce network partitioning, we also propose a partition handling routine to deal with network partitions to achieve high localization coverage. The major design goal of our work is to maximize localization coverage while keeping communication overhead minimum, thus achieving better energy efficiency. Major contributions of this paper are: (1) Two energy efficient relative localization techniques, and (2) A partition handling strategy that ensures localization of partitioned nodes.

## 1. Introduction

Recent years have witnessed a growing interest in the field of marine sciences. Extensive research has been conducted to explore and exploit environmental, ecological, economic, and defense-related aspects/potentials of world’s waters [1,2,3,4,5,6]. Underwater sensor networks (UWSNs) play a pivotal role in such studies by providing basic technical support for gathering, organizing, and reporting mission-critical data [7]. Besides some common characteristics between terrestrial and underwater sensor networks, such as large-scale deployment and energy constraints, UWSNs have some differences compared to their terrestrial counterparts [8,9,10]. For instance, unlike terrestrial sensor networks where radio waves are used as a medium of communication, UWSNs use acoustic waves predominantly [11,12]. Acoustic channel has limitations, such as scarce bandwidth and high probability of error [13]. Moreover, GPS that is predominantly used for localization of sensor nodes in terrestrial sensor networks cannot be used in UWSNs. This is due to the fact that radio waves used by GPS cannot penetrate more than a few meters inside water, thus rendering it useless for underwater applications [13]. In addition, underwater sensor nodes are prone to mobility with water currents, resulting in inconsistent network topology and errors in sensor node location estimates.

Most underwater applications require the sensed data to be tagged with location information. Data tagging is defined as labeling of sensed data with absolute position information of the location of collection of the data. Data tagging is important as sensed data without any information about its location will stand useless in most of the cases. Localization of sensor nodes is crucial for data tagging. For instance, in case of intrusion detection, location of intruding objects can be determined with the help of coordinates of sensor nodes deployed to detect intrusion. In case of marine life monitoring, sensor nodes observe movements of marine animals. Locations of sensor nodes observing movements can give insight into migratory patterns of animals. In case of underwater installation maintenance, locations of sensor nodes can help localize a malfunctioning part. Similarly, in case of oil spills and ocean trash, locations of sensor nodes can help determine the extent of affected area and concentrations of pollutants in different parts of the affected area. Besides, some protocols designed for underwater sensor networks require location information of sensor nodes in order to function properly. Some examples of, such protocols, can be found in the literature [14,15,16]. As all these (and many other) applications of UWSNs require location information of sensor nodes in order to be effective, therefore, localization of sensor nodes becomes an area of primary importance.

However, due to unavailability of GPS under water, localization of underwater sensor nodes becomes challenging. Constraints of the underwater environment, such as scarce bandwidth, high probability of error, and low propagation speed of acoustic waves [13,17,18], impose limitations to the design of localization algorithms. Mobility of sensor nodes and resultant partitioning of network into smaller disconnected sub-networks can affect localization ratios. In such partitioned networks, improvement in the localization ratio requires additional communication, thus reducing the energy efficiency of the network. The above-given constraints dictate the need for underwater localization mechanisms that should be able to: (a) achieve high localization coverage, (b) efficiently handle mobility related problems such as partitioning of the network, and (c) achieve high energy efficiency and minimize communication overhead.

**Figure 1:**
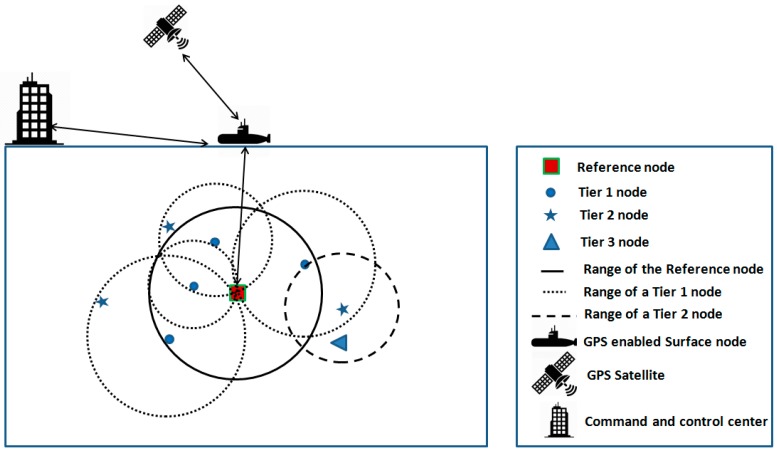
Network architecture of our proposed localization scheme.

Figure 1 shows the network architecture for our proposed localization scheme. Underwater sensor nodes can be divided into two types: (1) A reference node which has higher computational power, higher communication range and higher energy resource. The reference node gets its absolute coordinates through a Global Positioning System (GPS) enabled surface node. (2) Ordinary underwater sensor nodes. All underwater nodes can be divided into tiers. The reference node forms tier 0. It initiates the localization process by transmitting a beacon. All nodes that receive the beacon transmitted by the reference node constitute tier 1. All those that receive a beacon from any of tier 1 nodes form tier 2. Similarly, all those that receive a beacon from tier 2 nodes form tier 3. (It is important to note that tier formation is dependent on network topology and more importantly the order in which nodes access the channel after contention). The reference node updates its coordinates periodically by communicating with the surface GPS node. Moreover, the reference node periodically sends position information of all nodes to the surface GPS node which transmits the information to the command and control center. The surface GPS node is a propelled node that communicates with the reference node periodically to update reference nodes’ GPS coordinates.

We assume that all underwater nodes move freely with water currents and are equipped with a compass and necessary apparatus for time difference of arrival (TDoA) [19] calculation (i.e., every node is equipped with three non-collinearly positioned antennas). Reference node is assumed to be equipped with a movement tracking apparatus, such as a gyroscope. Movement tracking along with last received GPS coordinates enables the reference node to update its GPS coordinates while moving. All nodes are time synchronized and transmit with the same transmission power (P) which can be increased or decreased if a change in the transmission range (R) is required. We assume that relative positions of nodes do not change in the short run. This is a reasonable assumption as all nodes move within the same current. In order to compensate for multipath effect, we assume that all nodes employ the multipath compensation strategy proposed in Ref. [20]. Moreover, the transmission model is assumed to be omnidirectional. All nodes are deployed at equal depth, thus forming a 2D network. However, if nodes are deployed at different depth levels, depth information from pressure sensors can be used to translate a 3D problem to 2D.

In this work, we propose a two-stage localization scheme. In stage 1, all sensor nodes are localized relative to the reference node. This stage is executed periodically every t units of time. It is very important to note that stage 1 coincides with the sensing period. After every execution, the reference node has up-to-date locations of all sensor nodes relative to itself. These locations along with time information are stored by the reference node in a locally maintained table of relative location (TRL). If network partition is detected during stage 1, the reference node activates partition handling routine to access out of range nodes. In stage 2, absolute locations of all nodes are calculated using (a) table of relative locations of the reference node and (b) GPS coordinates of the reference node at time *t*, where *t* is the time when a particular node is localized relative to the reference node. It is important to note that stage 2 is carried out on a general-purpose computer. Therefore, costs (e.g., energy consumption, computational overhead) incurred by stage 2 are not considered in this work. It is important to mention here that this protocol is designed considering that applications require real-time information to be conveyed to C&C in order to be effective. Examples of such applications include surveillance and reconnaissance, intrusion detection, and disaster prevention. Since underwater sensor networks are scarce in energy, allowing every node to estimate and transmit location information to the surface GPS node (using high transmission power) may result in high energy consumption due to high transmission power. High transmission power not only consumes more energy but also creates higher levels of interference thus increasing contention that can result in retransmission and further energy consumption. Therefore, in our mechanism, we propose that relative positions of all nodes w.r.t. reference node are estimated. The reference node then sends its Table of relative locations (TRL) to the surface GPS node which then transmits it to C&C. C&C estimates absolute locations of sensors at time t using the absolute location of the reference node at time t and the relative position of the sensor node at time t.

The rest of this paper is organized as follows. Section 2 discusses related work. Section 3 explains the methodology of our protocol in detail. Section 4 presents the simulation setup. Section 5 describes the results and discussion. Section 6 presents the conclusion of this study.

## 2. Related Work

**Figure 2:**
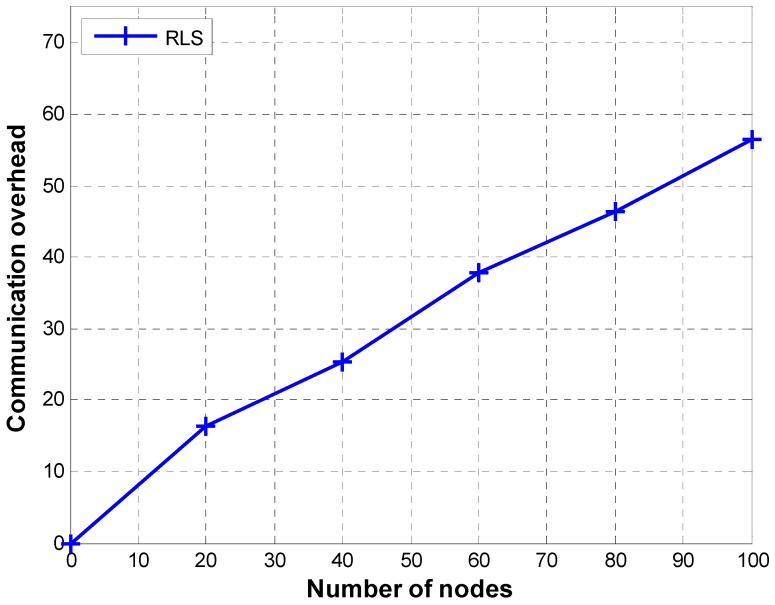
Communication overhead of Reverse Localization Scheme (RLS).

As a crucial part of underwater sensing missions, localization of nodes in underwater acoustic sensor networks has attracted serious attention of the research community. Many researchers have proposed sophisticated mechanisms to carry out energy efficient localization of underwater sensor nodes with low localization errors and good localization coverage. We will broadly categorize localization protocols into the following three categories:

1. **Nature of the computational algorithm**: Underwater location estimation algorithms may be categorized as centralized or distributed [21] depending on whether the localization is carried at a centralized location or whether every node finds its coordinate itself based on absolute coordinates of localized nodes. Each of these two types offers its own advantages and disadvantages [22]. For instance, the centralized technique relieves ordinary nodes of the computational burden. Therefore, it may save energy wastage due to computation. On the other hand, the centralized technique requires sensor nodes to communicate with anchor nodes. This results in higher communication overhead which translates to higher energy consumption.
  In a previous study [23], reverse localization scheme (RLS) has been proposed. RLS is an example of a centralized localization algorithm as the main localization algorithm is run at a centralized station. RLS carries out its operation in two stages: (1) transmission stage and (2) the geometric positioning stage that is run in a centralized node. In the transmission stage, messages are exchanged based on event-driven reporting. During the geometric positioning stage, the sink node runs a centralized location estimation algorithm to estimate locations of sensor nodes based on the information that it collects from anchor nodes.
  Area-based localization (ALS) [24] is another example of a centralized localization technique. In this scheme, anchor nodes transmit beacons periodically using different levels. Based on the ranges of anchor nodes, the network is divided into non-overlapping sub-regions. In this scheme, sensor nodes passively hear beacons transmitted by anchor nodes. On reception of beacons, sensor nodes pass on the information (which also includes power level used by the anchor node) to the sink node. As the sink node already knows the position of the anchor node, it can estimate coordinates of sensor nodes. This scheme does not require time synchronization. However, its drawback is high energy consumption due to high communication overheads.
  Multihop fitting localization approach (MFLA) [25] is an example of a distributed location estimation technique. This algorithm takes isolated unlocalized nodes into account. The authors consider a mobile UWSN in which nodes move with water currents. They may be isolated if they move too far to receive beacons. This method works by setting an intermediate node between the beacon node and the unlocalized node as a routing node to establish a path by a greedy algorithm. The multi-hop path is then fit into a straight line and the position of the node is estimated by trilateration.
2. **Mobility**: Mobility may affect the localization of sensor nodes. Therefore, localization algorithms can be classified based on whether they are designed for a mobile underwater sensor network or a static underwater sensor network.
  Silent positioning scheme [26] proposed in the UPS model is an example of a localization scheme that is designed for stationary underwater sensor networks. This scheme works for one-hop underwater networks. Location estimation is carried out with the help of four anchor nodes that can transmit beacons sequentially. Underwater ordinary sensor nodes do not send any localization messages. Thus, UPS is silent. The communication cost of UPS is low, making it more energy efficient. Silent Position scheme is a range-based scheme. It uses TDOA for range estimation. Therefore, it does not require time synchronization. A drawback of UPS is that it can localize only those nodes that are located inside the area enclosed by anchor nodes [21]. Moreover, positions of anchor nodes should be fixed and known to sensor nodes in advance [21].
  Localization with a directional beacon or LDB [27] is an example of a localization scheme which includes the notion of mobility. This technique employs an AUV as a mobile beacon. The AUV uses a directional transceiver. LDB is a range-free method. It is an improved version of UDB [28] as it assumes node deployment in 3D space, unlike UDB which assumes node deployment in 2D space. Sensor nodes passively listen to beacons from AUV and estimate their coordinates based on coordinates of the Autonomous vehicle at the time of entry and exit from the communication range of sensor nodes. As in LDB, nodes are localized based on beacons from the AUV. Thus, the accuracy of location estimates depends on the frequency of beacons from AUV. The accuracy of location estimation also suffers from both vertical and horizontal errors.
  A cluster-based localization scheme with partition handling for mobile underwater sensor networks [29] is another example of a localization scheme for mobile UWSN. This scheme works in two phases. In the first phase, a GPS-enabled node transmits a beacon which is received and forwarded by all nodes that receive the beacon. In stage 2, all nodes that could not receive beacon during stage 1 can send localization requests proactively. On reception of localization request, the GPS-enabled node sends a beacon. The main contributions of this technique are a clustering-based mechanism which minimizes energy consumption in stage 2 and a retransmission control mechanism which prevents unnecessary transmission.
3. **Algorithm Stages**: Localization schemes can be divided into two categories according to communication characteristics: (1) Single-stage schemes [30,31], and (2) multi-stage schemes [32,33,34]. In single-stage schemes, ordinary sensor nodes work in passive mode (i.e., they do not participate in localization activity except for activity related to their own localization). In case of single-stage protocol, ordinary sensor nodes do not act as reference nodes. Thus, they are not helpful for the localization of other ordinary nodes. However, in case of multi-stage protocols, an ordinary sensor node may act as a reference node as soon as it is localized. Unlocalized ordinary nodes can communicate with any of localized nodes to estimate their coordinates.
  The algorithm proposed in a previous study [35] is an example of a single-stage localization protocol. This algorithm uses a hyperbolic technique, normal distribution estimation error modeling, and calibration to localize a sensor node.
  The top-down positioning scheme proposed previously [36] is a multi-stage localization scheme. In this scheme, nodes are divided into three types: Anchor nodes that float on the water surface, reference nodes whose locations are known, and unlocalized nodes. Initially, as the localization process begins, nodes that fall within the range of anchor nodes are localized. Upon being localized, nodes can calculate confidence values and compare confidence values with confidence thresholds. If a node’s confidence value is higher than the confidence threshold, the node assumes the role of a reference node.
  TP-TSFLA [37] is another example of multi-stage localization schemes. It has 2-stages. Initially, during stage 1, reference nodes broadcast beacons. All those sensor nodes that receive beacons are localized using the received beacons. These newly localized nodes become reference nodes to help localize the remaining unlocalized node. In stage 2, all unlocalized nodes increase their transmission power to request beacon. Any localized node that receives the request responds with a beacon. The process of requesting localization beacon may continue until nodes are localized. This method may incur high communication overhead due to the fact that a particular unlocalized node may receive unnecessary beacons from a large number of reference nodes. Consequently, high communication overhead increases energy consumption.
  We will compare our technique with reverse localization scheme (RLS) [23]. The communication overhead incurred by RLS is shown in Figure 2. In case of RLS, for every node to be localized there are at least four transmissions, one by the sensor node and three by the surface reference nodes. Moreover, the number of transmissions increases if the signal transmitted by a sensor node is received by more than three reference nodes. The high communication overhead of RLS results in low bandwidth efficiency and high energy consumption.

## 3. Methodology

**Figure 3:**
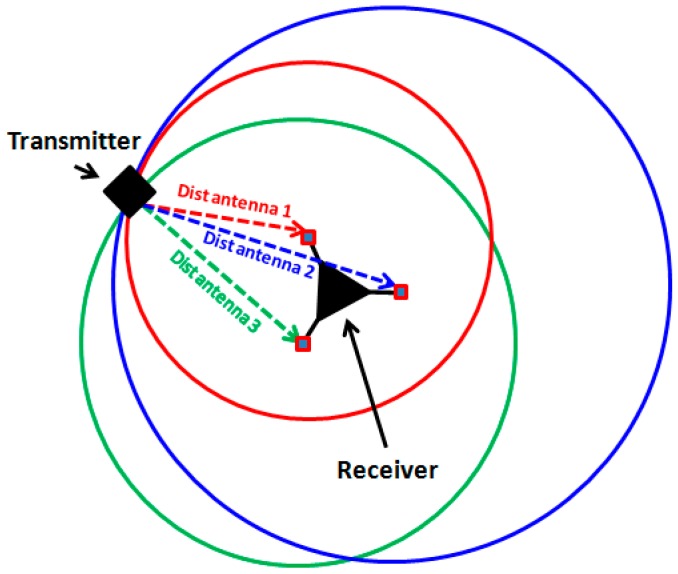
Use of trilateration for localization.

Figure 3 shows the use of trilateration for location estimation of nodes. All nodes are equipped with three non-collinearly positioned antennae. The incoming signal is received by the three antennae. Each antenna estimates its distance from the sender. This information is then processed centrally by the receiving node which applies trilateration to estimate the relative location of the sender with respect to itself.

### 3.1. Protocol 1.1

As stated earlier, our scheme works in two stages. The first stage is called relative localization stage. It is executed periodically. Its execution cycles coincide with sensing cycles so that positions of sensor nodes at the time of sensing can be estimated. Relative localization stage deals with the estimation of relative positions of underwater sensor nodes with respect to the reference node. The estimated relative position information is transmitted to the command and control center (C&C) through the surface GPS node. The C&C runs the second stage (called absolute localization stage) to calculate absolute coordinates of sensor nodes at time t using the absolute position of the reference node at time t and relative position of sensor node w.r.t. the reference node at time t, where t is the time when a particular node is localized relative to the reference node.

#### Relative Localization Stage

Relative localization stage can be divided into two parts: Downstream/forward propagation of a beacon from the reference node to ordinary nodes and backward propagation of local tables of relative locations (TRLs) from ordinary sensor nodes to the reference node.

#### Beacon Propagation

**Figure 4:**
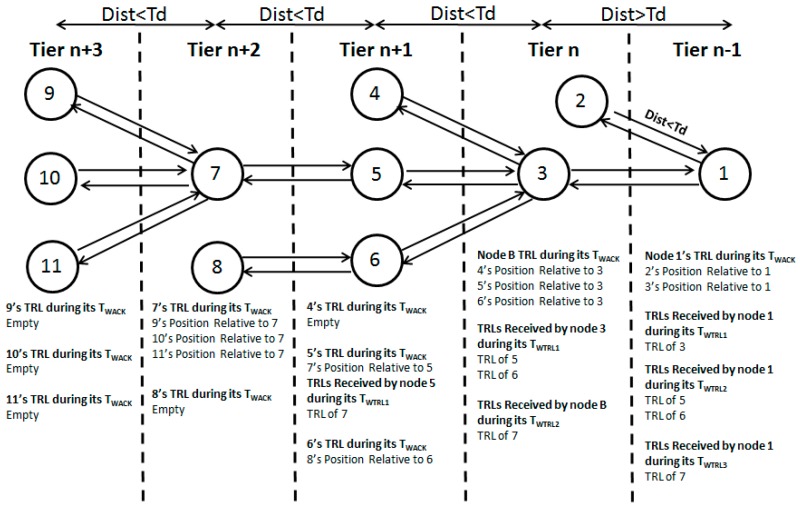
Beacon propagation.

Figure 4 shows the propagation of beacon in the network. The reference node (node 1) initiates the relative localization stage by transmitting a beacon (B) which propagates down to edges of the network traversing various paths. Every node y on the way that receives beacon (B) responds by taking the following set of actions.

**Figure 5:**
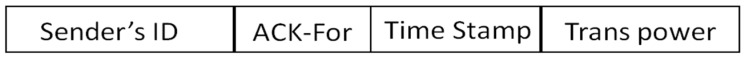
Beacon format.

- It calculates its distance from the sender (node x) using RSSI and saves the calculated distance for future use.
- If the distance is more than the (default range − guard value), node y adjusts its transmission power to yield a transmission range that is equal to the (CalculatedDistance+GuardValue) and retransmits B with appropriate header values as shown in Figure 5. (Guard value is used to compensate for variation in range due to channel impairments). Otherwise, node y retransmits B using default transmission power. B serves as ACK for the sender of the beacon (node x) and as a beacon for those nodes in node y’s range that did not receive a beacon from any other node. The “ACK-For” field in B contains ID of the node that should consider B as ACK (i.e., node x). Upon reception of ACK, node x calculates the position of node y relative to itself (i.e., node x) using TDoA.
- Calculate T_WACK_ and go into wait state for the duration of T_WACK_. T_WACK_ is the time period that a node that transmits a beacon will wait for ACKs in response to the beacon (B) is transmitted.

Beacon propagation can be further elaborated with the help of Figure 4 as follows. The reference node (node 1) initiates stage 1 by transmitting a beacon. Node 2 and node 3 receive the beacon and calculate their distance from node 1 using RSSI. Assuming the distance of node 2 from node 1 is less than the (default range − guard value), node 2 retransmits B with default transmission power. As there is no other unlocalized node in node 2’s range, the beacon propagation on this path stops here. However, Node 1 receives B (as ACK) and calculates the relative position of node 2 w.r.t. itself using TDoA. Unlike node 2, node 3’s distance from node1 is bigger than the default range. Therefore, node 3 adjusts its transmission power according to its distance from node 1. This will ensure that B from node 3 can reach node 1. On reception of B, node 1 calculates relative positions of node 3 w.r.t. itself. As nodes 4, 5, and 6 fall within the range of node 3, they also receive B transmitted by node 3. As already stated, a retransmitted B can act as a beacon for those nodes in sender’s range that have not received a beacon so far. Therefore, nodes 4, 5, and 6 will consider B as beacon and follow the same steps as those taken by nodes 2 and 3 on reception of beacon from node 1. These steps will be followed by every node that receives B as a beacon, thus propagating localization beacon to edges of the network. Similarly, on reception of B’s from nodes 4, 5, and 6, node 2 will follow the same steps as those taken by node 1 on reception of B from nodes 2 and 3. These steps will be followed by every node that receives B as acknowledgement.

#### Backward Propagation of TRLs

**Figure 6:**
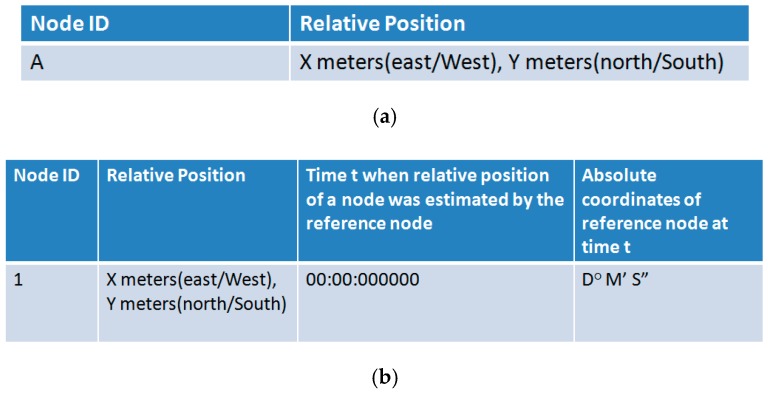
(**a**) TRL of an ordinary node. (**b**) TRL of reference node.

When a node receives B as ACK for its beacon, it calculates the relative position of the sender w.r.t itself using TDOA and saves it in a locally maintained table of relative locations or TRL (Figure 6a,b). Thus, every upstream node that transmits a beacon can calculate relative positions of all those downstream nodes that send ACKs (i.e., B) in response to its beacon. For instance, in Figure 4, node 1 calculates relative positions of nodes 2 and 3, node 3 calculates relative positions of nodes 4, 5, and 6, and nodes 5 and 6 calculate relative positions of nodes 7 and 8. At this stage, none of these nodes knows relative positions beyond its neighbors in the next tier. For instance, node 1 knows relative positions of only nodes 2 and 3, but not relative positions of any other nodes. However, as stated earlier, our objective is to localize every node relative to the reference node. For this, we need a mechanism to move the locally maintained TRL’s to the reference node (node 1). Then the reference node can apply lemma1 (Equations (1) and (2)) to estimate positions of all nodes relative to itself. The following text explains the backward propagation of TRLs from ordinary sensor nodes to the reference node.

Any ordinary node R of tier n that retransmitted beacon that it received from some node Q of tier n − 1 waits for ACKs for a certain threshold period of time called T_WACK_ (TWACK is set to a multiple of the round-trip time between sender and receiver). During T_WACK_, if node R receives ACK/TRL from any node S of tier n + 1, it calculates positions of node S (or the positions of nodes in TRL if TRL is received) relative to itself (i.e., node R) and saves it in its table of relative locations. At the end of its T_WACK_, node R’s TRL contains IDs and relative locations of all those nodes S (and the nodes in node S’s TRL (if TRL is sent)) that sent an ACK in response to its beacon. Figure 4 shows the TRLs of all nodes at the end of their respective T_WACK_ (for the sake of simplicity, we assume that the nodes win contention in the sequence shown in Figure 4. However, in real life scenarios, any node may win contention which will change the order in which ACKs/TRLs are propagated upstream). It is important to note that T_WACK_ is set only once after the transmission of a beacon.

At the end of its respective T_WACK_, every Node R of tier n sends its TRL to the node Q in tier n − 1 from which it received the beacon. It also sets timer T_WTRL_1 (similar to T_WACK_, T_WTRL_’s are also set to multiples of the round-trip time between sender and receiver. T_WTRL_1 is the time node R will wait for TRL(s) from all nodes S(i) of tier n + 1 which received beacon from node R. At the end of T_WTRL_1, node R has TRLs from all nodes S(i) in tier n + 1 which received beacon from node R. Node R combines all received TRLs and sends them to node Q (in tier n − 1) from which it received the beacon. Node R also sets its second T_WTRL_ (i.e., T_WTRL_2) as soon as T_WTRL_ 1 expires. During T_WTRL_2, node R waits for TRLs from those nodes in tier n + 2 which has received a beacon that passed through node R. At the end of T_WTRL_2, node R has TRLs of all its two hops neighbors in tier n + 2. Node R combines all received TRLs and sends them to node Q in tier n − 1. Moreover, it again calculates its next waiting time (i.e., T_WTRL_3) as soon as T_WTRL_2 expires. During T_WTRL_3, it waits for TRLs from its three hop neighbors in tier n + 3. This process continues until node R does not receive any TRL.

The above-mentioned process runs in every node and stops when a node does not receive ACK or TRLs during its T_WACK_ or T_WTRL_, respectively. This method ensures that TRLs of every node are propagated back to the reference node on the same path (in reverse order) as that of beacon propagation. Figure 4 shows TRLs received by nodes during their T_WTRL_’s.

Just like other nodes, the reference node also has waiting times (i.e., T_WACK_) during which it waits for ACKs in response to the beacon it transmits and T_WTRL_’s during which it waits for TRLs from different tiers. If the reference node does not receive any ACK or TRL during a particular waiting time, its TRL (Figure 6b) is not complete (the reference node has a list that contains IDs of all nodes in the network). After its T_WACK_ and each of its T_WTRL_, the reference node compares its TRL with the list of all nodes. The two lists match if all nodes have been localized relative to the reference node. Otherwise, if there is a mismatch, it assumes that the network is partitioned. Therefore, it calls “partition handling” routine which involves increasing the transmission power of certain nodes. Partition handling is explained at the end of Section 3.1.

Table 1 present a summary of how a node will react during and at the end of its T_WACK_. Table 2 presents a summary of how a node will react during and at the end of its T_WTRL_’s.

#### Multi-Hop Relative Localization

All tier 1 nodes (i.e., those nodes that receive beacon directly from the reference node) are localized relative to the reference node using TDoA based on ACKs received from them. However, lemma 1 is used for nodes whose ACKs cannot reach the reference node directly (i.e., node in tier 2 and above).

**Lemma** **1.** *Assume three nodes A, B and C. Node A can estimate the position of node C relative to node A if it knows the position of node B relative to Node A and the position of node C relative to node B using Equations (1) and (2).*

X_ac_ = X_ab_ + X_bc_ (1)

Y_ac_ = Y_ab_ + Y_bc_ (2)

**Figure 7:**
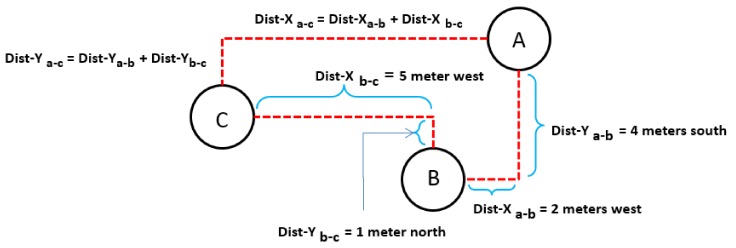
Relative localization of nodes in tier 2 and above w.r.t. the reference node.

Lemma 1 can be explained as follows. In Figure 7 we assume that node A knows the position of node B relative to itself (i.e., node A) and node B knows the position of node C relative to itself (i.e., node B). These relative positions are found using TDoA and trilateration. The objective is for Node A to find the position of node C relative to itself. All nodes are equipped with compass using which they can divide the area around them into four quadrants. Using the relative position information, node A can tell how many meters away node B is in any particular direction. For instance node B can tell that node C is located one meter to the north (which can translate to +1 *y* axis when node B assumes itself on (0,0)) and five meters to the west (which can translate to −5 on *x* axis when node B assumes itself on (0,0)) of node B. Similarly, from its TRL node A can tell that node B is two meters to node A’s west (which can translate to −2 on *x*-axis when node A assumes itself on (0,0)) and four meters to node A’s south (which translates to −4 on *y*-axis when node A assumes itself on (0,0)). Now the position of node B relative to node A on a Cartesian plane that is centered at node A is (−2, −4). Similarly, the position of node C relative to node B on a Cartesian plane that is centered at node B is (1, −5). Node B sends the information that it has about the relative position of node C to node A (i.e., node B tells node A that node C is located one meter to the north and five meters to the west of node B. Now node A has two pieces of information:

Node B is located two meters to the west and four meters to the south of node A or at position (−2, −4) with respect to node A when node A is at (0,0).

Node C is located one meter to the north and five meters to the west of node B or at position (1, −5) with respect to node B when node B is at (0,0). This information is sent by node B to node A.

Using these two pieces of information in Lemma 1, node A can estimate the position of node C relative to itself.

#### Waiting Period

We will define two types of waiting periods: T_WACK_ and T_WTRL_.

**T_WACK_:** T_WACK_ (Equation (3)) is defined as the time period any node x will wait for ACKs after transmission of a beacon (B).

(3) $$T_{\mathrm{WACK}}=\beta \times 2\times \frac{R}{1500}+{\mathrm{Prc}}_{d}+\;\mathrm{Guard}\;\mathrm{Time}$$

where β which is a function of network sparsity, regulates the waiting time _TWACK_. R is the transmission range of node x, 1500 represents the propagation speed of sound in water in meters per second, Prc_d_ is processing delay and Guard time is used to compensate for transmission delay variations due to channel asymmetry.

**T_WTRL_:** T_WTRL_ is defined as the time period a node will wait for TRLs after the end of its T_WACK_ or its previous T_WTRL_. During any particular _TWTRL_, the waiting node receives TRL’s of the nodes in next tier (or tiers beyond next tier) Figure 4 shows TRLs of every node at the end of their respective T_WTRL_’s. For the sake of simplicity, we assume that the nodes win contention in the sequence shown in Figure 4. However, in real life scenarios, any node may win contention which will change the order in which TRLs are propagated upstream.

T_WTRL_’s for any node can be calculated using Equation (4).

(4) $$T_{\mathrm{WACK}}=\alpha \times 2\times \frac{R}{1500}+{\mathrm{Prc}}_{d}+\;\mathrm{Guard}\;\mathrm{Time}$$

where α which is a function of network sparsity, regulates the waiting time T_WTRL_.

**Figure 8:**
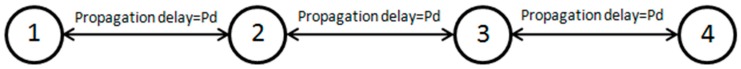
Calculation of T_WTRL_.

Table 3 illustrates beacon and TRL propagation in the context of waiting times. It also shows actions taken by each node on expiry of their T_WACK_ or T_WTRL_. For the sake of brevity, in Figure 8, we assume only one node per tier. Every node is located on the edge of the transmission range of the node in the previous tier. For the sake of simplicity of explanation, we ignore guard value in Table 3. Moreover, α and β are assumed to be one.

#### Handling Network Partitions

**Figure 9:**
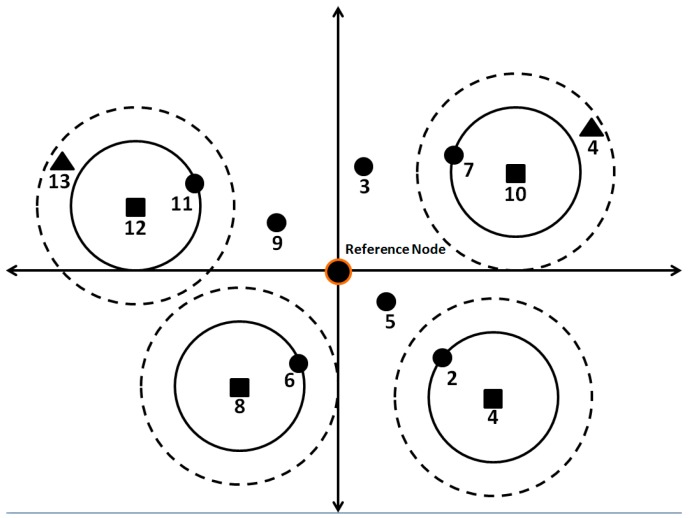
Partition handling.

If by the end of a certain waiting time of the reference node (T_WACK_ or T_WTRL_) no ACK or TRL is received by the reference node while there are still one or more nodes that remain to be localized, then the network is assumed to be partitioned. In such a case, the reference node will activate partition handling mechanism which works as follows:

The farthest localized nodes (localized relative to the reference node) in each of four quadrants of the reference node will double the default transmission power to search for the missing node(s). Figure 9 presents a scenario to elaborate on this point. In Figure 9 we assume that all nodes except nodes 4 and 13 have been already localized using initial localization steps as explained so far. Node 4 and 13 are partitioned nodes i.e., they are out of the communication range of all the other nodes and therefore could not be localized. In order to localize nodes 4 and 13, node 1 selects the farthest nodes (represented by squares) in each quadrant. Moreover, the four selected nodes transmit beacon with doubled transmission power so that that the beacon can reach the partitioned nodes. The dotted circles show increased range. With increased ranges, nodes 4 and 13 receive beacons from nodes 10 and 12, respectively. On reception of beacon, nodes 4 and 13 respond with ACKs (B) to nodes 10 and 12, respectively, as explained previously. From here on, the same process of hop by hop backward TRL transmission will be followed as explained in Section 3.1.

Reasons for selecting four nodes (one in each quadrant) in order to localize the missing nodes are shown below:

1. The missing nodes can be in any direction relative to the reference node.
2. The reference node can be located anywhere in the localized part of the network. For instance, it can be located somewhere in the center or in any direction far away from the center of the localized part of the network. If the GPS-enabled node is somewhere near the center, it will have to increase its range much more as compared to a node on the border of the localized part of the network in order to reach a missing node. If it is located far from the center in any particular direction and the missing nodes are located in the opposite direction, the reference node will have to increase its range even more compared to the case when it is situated in the center, thus wasting a lot of energy. Therefore, in order to save energy, we choose one farthest node in each quadrant. This ensures that unlocalized nodes are accessed with a much smaller increase in range, thus saving energy. Moreover, as the task is divided, the energy dissipation is fair. To make it fair, if a node has been used once to locate missing nodes, it will not be used again (for a certain number of times) even if it is the farthest in a quadrant. In such a case, the second farthest node will be used to locate missing nodes.

### 3.2. Protocol 1.2

Protocol 1.2 is the same as protocol 1.1 except for a different technique for backward propagation of TRLs. As can be seen in Table 2, the backward propagation of TRLs to reference nodes requires many transmissions. The number of transmissions can increase even more if the network has a higher number of nodes and many tiers. The increase in the number of transmissions not only increases communication overhead, but also increases contention, thus increasing overall convergence time and energy consumption. Therefore, we propose Protocol 1.2 which is a more efficient technique to propagate TRLs back to reference nodes.

In this technique, every node is responsible for sending only its own TRL to the reference node. Unlike the previous technique, in this technique, nodes will not act as relays for TRLs of upper tier nodes. Instead, nodes in every tier will calculate their distance from the reference node and adjust their transmission powers to yield a range equal to or greater than the calculated distance. In this technique, a tier n node that exists on a path that consists of m tiers will have to make only one TRL transmission (irrespective of the number of tiers) as opposed to m-n TRL transmissions in case of the first technique. In order to calculate its distance from the reference node, a node that wants to send a TRL needs to know the relative position of the reference node w.r.t. itself. The previous technique does not include this calculation. However, in this technique, we make it mandatory for every node x belonging to any tier n to calculate its position relative to the reference node and add this information to the beacon it will transmit. On reception of this beacon, node y in tier n + 1 will calculate the relative position of the sender (i.e., node x w.r.t. itself). Using the calculated relative position and the relative position of the reference node w.r.t. node x, node y will calculate the relative position of reference node w.r.t. itself using Lemma 1. Based on the distance between itself and the reference node, the node adjusts its transmission power and sends TRL directly to the reference node adding ID of the reference node in the destination field, thus avoiding many unnecessary transmissions.

### 3.3. Stage 2: Absolute Localization

Absolute positioning phase is carried out on general purpose computers in C&C. The computer is assumed to contain per degree distance information at any latitude or longitude. This assumption is reasonable as this step is carried out on general purpose computers and therefore there are no storage or processing power limitations.

Assume that at a particular time t, the reference node A is located at x degrees latitude and y degrees longitude. We can determine the absolute coordinates of a node B which is located *f* meters to the east/west and *g* meters to the north/south of the node A by the following calculations.

It should be noted that the table of relative locations of A mentions distances of nodes relative to node A along with directions. For instance, table of relative locations of A may mention that node B is located X km to the east and Y km to the south of node A.

**Figure 10:**
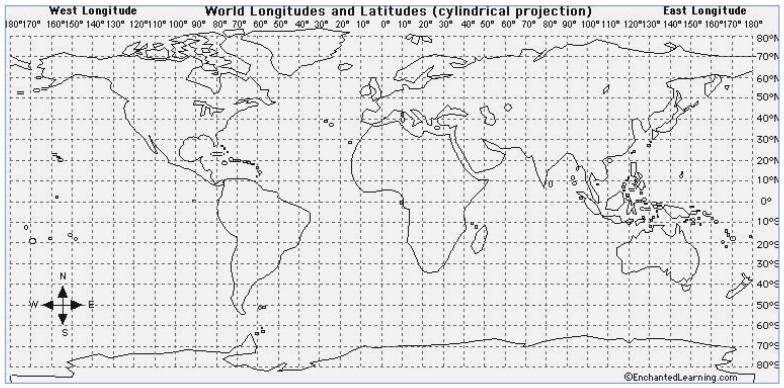
World longitudes and latitudes (cylindrical projection) [38].

Using the map representation given in Figure 10, there can be three cases each for latitude and longitude calculation.

#### 3.3.1. Latitude Calculation:

Node A is located in the southern hemisphere and node B is located south of node A or Node A is located in the northern hemisphere and node B is located north of node A. In this case, the latitude of B can be calculated using the following calculations.

1. Node A is located in the southern hemisphere and node B is located south of node A or Node A is located in the northern hemisphere and node B is located north of node A. In this case, the latitude of B can be calculated using Equations (5) and (6).
  LatB = distYAB° + LatA(5)
  whereas
  distYAB° = DY_AB(TRL)_/111.2; (6)
  DY_AB(TRL)_ is obtained from Node A’s TRL. It is the distance between points A and B along Y axis in kilometers. In this case direction in LatB remains the same as the direction of LatA, i.e., both the points are located in the same hemisphere.
  Example:
  Node A’s absolute position = 20° S 150° E
  Node B is located 1668 KM to the South of A.
  distYAB° = 1668/111.2 = 15°
  LatB = 15° + 20° = 35° S
2. Node A is located in the southern hemisphere and node B is located north of node A or Node A is located in the northern hemisphere and node B is located south of node A. whereas the distance (in degrees) between point A and B along latitude (i.e., distYAB°) is less than LatA. In this case, the latitude of B can be calculated using Equation (7).
  LatB = LatA − distYAB°(7)
  In this case, the direction of LatB remains the same as the direction of LatA, i.e., both the points are located in the same hemisphere.
  Example:
  Node A’s absolute position = 20° S 150° E
  Node B is located 1668 KM to the North of A.
  distYAB° = 1668/111.2 = 15
  LatB = 20 − 15 = 5° S
3. Node A is located in the southern hemisphere and node B is located north of node A or Node A is located in the northern hemisphere and node B is located south of node A. Whereas the distance (in degrees) between point A and B along latitude (i.e., distYAB°) is greater than LatA. In this case the latitude of B can be calculated using Equation (8).
  LatB = distYAB° − LatA(8)
  It is important to mention that in this case direction in LatB is opposite of A, e.g., if LatA is in the south then LatB is in the north.
  Example
  Node A’s absolute position = 10° S 150° E
  B is located 1668 KM to the North of A at 150° E
  distYAB° = 1668/111.2 = 15
  LatB = 15 − 10 = 5° N

#### 3.3.2. Longitude Calculation:

1. Node A is located in eastern hemisphere and node B is located east of node A or Node A is located in western hemisphere and node B is located west of node A. In this case the longitude of B can be calculated using Equations (9) and (10).
  LongB = distXAB° + LongA(9)
  whereas
  distXAB° = DX_AB(TRL)_/(111.2 × cos(lat(A))); (10)
  In this case, the direction of LongB remains the same as the direction of longA, i.e., if LongA is east then Long B is also east or if LongA is west, then LongB is also west.
  Example:
  Node A’s absolute position = 20° S 50° E
  B is located 2403.36 KM to the East of A at 20° S
  distXAB° = 2403.36/(111.2 × cos(20)) ≈ 23°;
  LongB = 23° + 50°= 73° E
2. Node A is located in Eastern hemisphere and Node B is located west of Node A or Node A is located in western hemisphere and node B is located east of node A. Whereas the distance (in degrees) between point A and B along longitude (i.e., distXAB°) is less than LongA. In this case the longitude of B can be calculated using Equation (11).
  LongB = LongA − distXAB°(11)
  In this case, the direction of LatB remains the same as the direction of LatA, i.e., both the points are located in the same hemisphere.
  Example:
  Node A’s absolute position = 20° S 50° E
  B is located 2403.36 KM to the west of A at 20° S
  distXAB° = 2403.36/(111.2 × cos(20)) ≈ 23°;
  LongB = 50° − 23°= 27° E
3. Node A is located in Eastern hemisphere and Node B is located west of Node A or Node A is located in western hemisphere and node B is located east of node A. Whereas the distance (in degrees) between point A and B along longitude (i.e., distXAB°) is greater than LongA. In this case, the longitude of B can be calculated using Equation (12).
  LongB = distXAB° − LongA(12)
  Its important to mention that in this case direction in LongB is opposite of A, e.g., if LongA is east, then LongB is west
  Example:
  Node A’s absolute position = 20° S 10° E
  B is located 2403.36 KM to the west of A at 20° S
  distXAB° = 2403.36/(111.2 × cos(20)) ≈ 23°;
  LongB = 23° − 10°= 13° W

## 4. Simulation Setup

### 4.1. Underwater Energy Consumption Model

Equation (13) represents path loss of the underwater acoustic channel. It is widely used in the literature [39]. In Equation (13), R, f, and SL represent transmission range, frequency (measured in kHz), and spreading loss, respectively. In the literature, commonly used values of SL are one for cylindrical spreading, two for spherical spreading, and 1.5 for practical spreading. A (f) which is absorption coefficient is calculated using Equation (14) in dB/km.

(13) $$\xi \left(R,f\right)=R^{\mathrm{SL}}\times A{\left(f\right)}^{R}$$

(14) $$10\;\mathrm{logA}\left(f\right)=0.11\frac{f^{2}}{1+f^{2}}+\;44\;\frac{f^{2}}{4100+f^{2}}+\;2.75\;\times 10^{-4}f^{2}+0.003$$

Generally, when f is above a few hundred Hertz, we use Equation (14). However, when f is smaller, Equation (15) [29] is mostly used.

(15) $$10\mathrm{logA}\left(f\right)=0.002+0.11\;\frac{f^{2}}{1+f^{2}}+0.011f^{2}$$

Equation (16) [39] calculates the power consumed when a packet is transmitted over distance R (in meters) using frequency f (in kilo Hertz):

(16) $$\rho_{\mathrm{db}}\left(d,f\right)=\Gamma \left(f\right)\gamma \left(d,f\right)\beta \left(f\right)\omega$$

where Γ(f), β(f), and ω represent power spectral density of noise, bandwidth around the center frequency f, and the target SNR at the receiver, respectively.

Equation (17) [18] is used to convert acoustic power $\rho_{\mathrm{db}}\left(R,f\right)$ in decibels to electrical power $\rho_{\mathrm{watts}}\left(R\right)$ in Watts:

(17) $$\rho_{\mathrm{watts}}\left(R\right)=\rho_{\mathrm{db}}\left(R,f\right)\times 10^{-17.2}/\psi$$

In Equation (17), ψ represents the efficiency of electric circuitry whereas $10^{-17.2}$ is the conversion factor.

Equation (18) [40] calculates total power consumption Ω:

(18) $$\Omega =\rho_{\mathrm{watts}}\left(R\right)+P_{R}$$

where Ω represents total power required for transmission over distance R, $\rho_{\mathrm{watts}}\left(R\right)$ represents transmit power, and $P_{\mathrm{Rc}}$ represents the fixed overhead incurred for receiving data. All these values are measured in watts.

Equation (19) calculates the amount of energy consumed for transmission of a packet of size ω bits with a transmission rate of TR:

(19) $$\varepsilon =\Omega \times \frac{\omega }{\mathrm{TR}}$$

### 4.2. Parameters Setting

We used Matlab R2016b to simulate our protocol. Table 4 shows simulation parameters. Nodes are assumed to move freely with currents. However, we also assume that during a localization period, their positions relative to each other do not change. This assumption is reasonable as all nodes move within the same current.

## 5. Results and Discussion

### 5.1. Fixed Simulation Area

In this section, we will run simulations for different numbers of nodes (i.e., 20, 40, 60, 80, and 100) and different numbers of partitions (i.e., one, two, three, and four partitions) over a simulation area of 1000 × 1000 m. We will compare our protocol 1.1 (shown as P1) and protocol 1.2 (shown as P2) with reverse localization scheme [23] (shown as RLS). All the protocols use distributed coordinated function (DCF) based channel access protocol as a channel access technology.

#### 5.1.1. Communication Overhead

**Figure 11:**
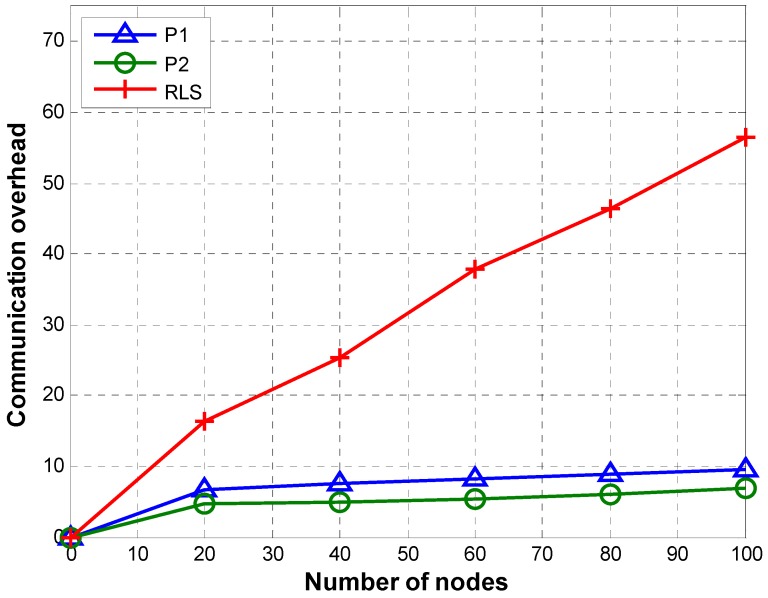
Communication overhead.

Figure 11 shows a comparison of communication overhead of P1, P2, and RLS. Communication overhead is computed using Equation (20) in which Г stands for the total number of transmissions required to localize L number of nodes:

(20) CO=ΓL

As explained earlier P1 and P2 can handle network partitioning efficiently. In both P1 and P2, every node except nodes selected by the reference node for retransmission can transmit beacon only once. In case of P1, TRLs are transmitted by those nodes that fall on the path from the reference node to the node whose TRL is being transmitted. Even though P1 achieves small communication overhead compared to RLS, it still has redundant transmissions of TRLs. P2 reduces the number of TRL transmissions by configuring only the TRL owner node to send TRLs directly to the reference node. This decreases the number of TRL transmissions significantly, resulting in decreased communication overhead in case of P2.

In case of P2, the number of per node beacon transmissions remains almost the same irrespective of the total number of nodes. Except for the farthest nodes selected by the reference node for retransmission (to resolve network partitioning), every node transmits beacon only once. Moreover, the number of TRL transmissions and the difference between the number of TRL transmissions for a different number of total nodes is small. Such a small difference in the total number of transmissions per node results in almost similar communication overhead for different numbers of nodes (i.e., 20, 40, 60, 80, and 100).

In case of P1, the number of per node beacon transmissions is similar to P2. However, an increase in the number of TRL transmissions due to an increase in the number of nodes results in a slight increase in communication overhead as the number of nodes increases from 20 to 100.

RLS shows an increase in communication overhead with an increase in the total number of nodes. This is due to the fact that the number of reference nodes increases with the number of sensor nodes (reference nodes are 12% of sensor nodes [23]). Therefore, more reference nodes will transmit localization messages to sink node when the number of sensor nodes is bigger. Moreover, as we use DCF for channel contention, each of the anchor nodes transmits at least four messages for successfully delivering a packet to command and control center. As many anchor nodes may receive the localization beacon/packet transmitted by a sensor node, multiple transmissions (i.e., RTS, CTS, beacon, and ACK) by each receiving anchor node create a heavy communication overhead. The communication overhead increases with increase in the size of the network as more anchor nodes are deployed for a bigger network.

#### 5.1.2. Energy Consumption

We assume that all nodes are equipped with Evo Logic’s S2CR 48/47 acoustic modem [41]. Table 5 shows power consumption by the modem for transmitting over different ranges.

**Figure 12:**
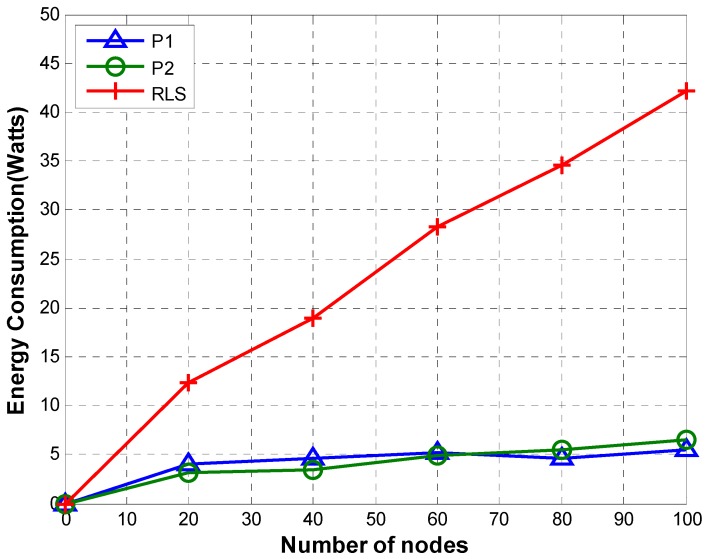
Energy consumption.

Figure 12 shows a comparison of average energy consumption of P1, P2, and Reverse Localization Scheme (RLS). Average energy consumption is computed using Equation (21).

(21) $$\xi =T_{EC}/\eta$$

where ξ, $T_{EC}$, and η represent average energy consumption, total energy consumed by the localization procedure, and the total number of nodes, respectively.

The high communication overhead of RLS due to transmission by multiple anchor nodes results in high energy consumption of RLS. Compared to RLS, P1 and P2 consume much less energy due to lower communication overhead. Although the communication overhead of P2 is smaller than that of P1, their energy consumption is similar. This is due to the fact that TRLs transmitted in case of P2 are transmitted directly to the reference node using high transmission power. Whereas in case of P1, TRLs are routed from the sender to the reference node hop by hop. Every node along the path uses only a small transmission power that is enough to reach the next node on the path.

#### 5.1.3. Localization Error

**Figure 13:**
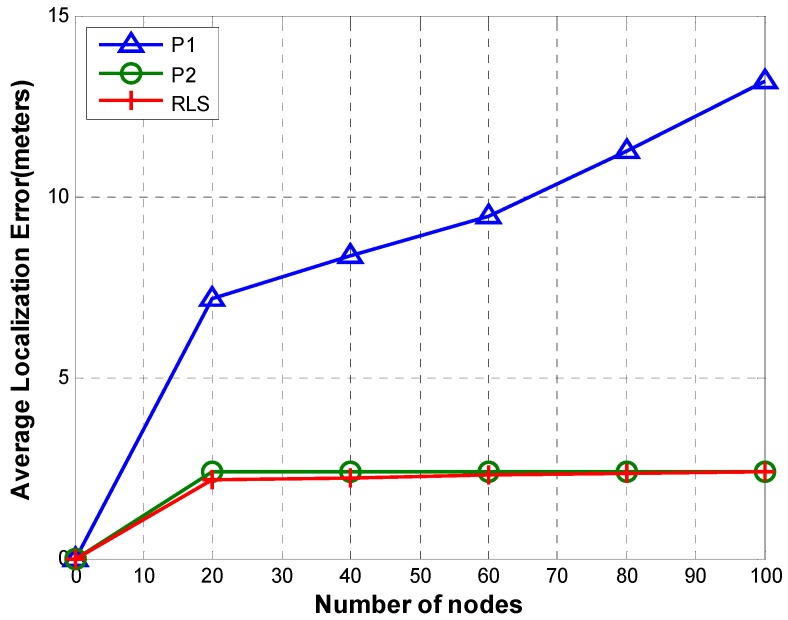
Localization error.

Figure 13 shows a comparison of localization error of RLS, P1, and P2 for different number of nodes (20, 40, 60, 80, and 100).

In case of both P1 and P2, nodes are localized relative to the reference node. In P1, TRLs are routed to the reference node passing through all tiers between the TRL owner and the reference node. This results in error accumulation as TRL passes through hops. Therefore, the accumulated error increases as the number of tiers increases. On the contrary, as shown in Figure 13, P2’s localization error not only is very low but also has a very small variation as the number of nodes increases. This is due to the fact that in P2, TRLs are sent directly to the reference node with higher transmission power instead of routing TRL through multiple tiers along the path from TRL owner to the reference node. In other words, error accumulation is avoided by cutting out all hops. RLS shows the smallest localization error. This is due to the fact that every sensor node can access reference nodes directly. Therefore, there is no error accumulation.

#### 5.1.4. Localization Coverage

**Figure 14:**
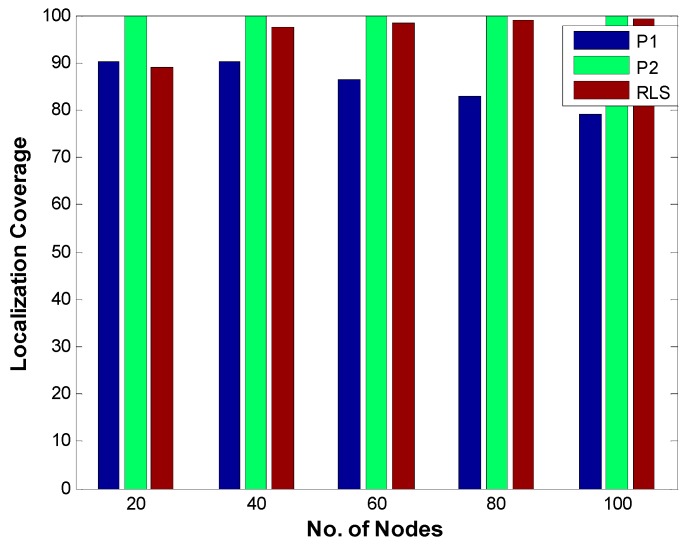
Localization Coverage.

Figure 14 shows a comparison of localization coverage of RLS, P1, and P2 for different number of nodes (20, 40, 60, 80, and 100).Localization coverage is computed using Equation (22) in which L and η represent the number of localized node and the total number of nodes, respectively.

(22) LC=Lη × 100

A node is considered unlocalized if it fails to receive a beacon or if its localization error is above a certain threshold value. Due to very low localization error, P2 achieves the maximum localization coverage whereas P1 which has higher localization error compared to P2 achieves smaller localization coverage. In case of RLS which has low localization error, the main determinant of localization coverage is the accessibility of sensor nodes to reference nodes. For the smaller number of nodes (which means the smaller number of anchor nodes as the number of anchor nodes is a specific smaller percentage of the number of sensor nodes), the localization coverage is small. However, as the number of anchor nodes increases due to an increased number of sensor nodes, the localization ratio improves due to an increased probability that a sensor node will be able to access enough number of reference nodes.

### 5.2. Sparsity

In this section, we will compare communication overhead, energy consumption, localization error, and localization coverage of P1, P2, and RLS for different network sparsity. Using normal distribution, 50 nodes are distributed over network areas of 500 × 500, 1000 × 1000, 1500 × 1500, and 2000 × 2000.

#### 5.2.1. Communication Overhead

**Figure 15:**
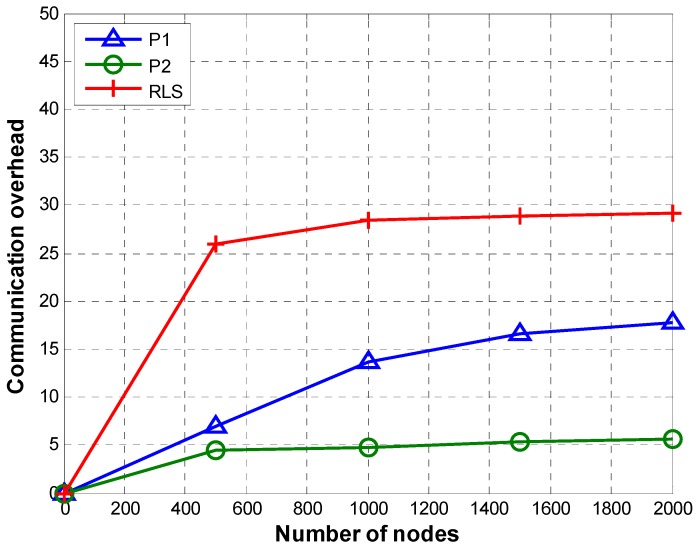
Communication overhead (sparsity).

Figure 15 shows CO of P1, P2, and RLS. P2 has the lowest communication overhead which remains almost constant through different networks’ sparsity. The reason for high communication overhead RLS is that in RLS besides contention based overhead incurred for transmitting a localization message to anchor nodes, each anchor node that receives a beacon transmits at least four messages for forwarding the localization message to the command and control center. Compared to P1, P2 has lower communication overhead. This is because in P2, TRLs are sent directly to the reference node whereas, in case of P1, every node X that falls on the path from the TRL owner to the reference node acts as a relay and forwards the TRL upstream towards the reference node, thus increasing communication overhead due to multiple relay transmissions.

The communication overhead of P2 remains almost constant irrespective of network sparsity. This can be explained as follows: (1) Every node transmits beacon once irrespective of the sparsity, and (2) in P2, the difference in the number of TRLs transmitted for different network sparsity values is very small. Thus, the same number of beacon transmission and the negligible difference in the number of TRL transmissions results in almost constant communication overhead at different values of network sparsity.

#### 5.2.2. Energy Consumption

**Figure 16:**
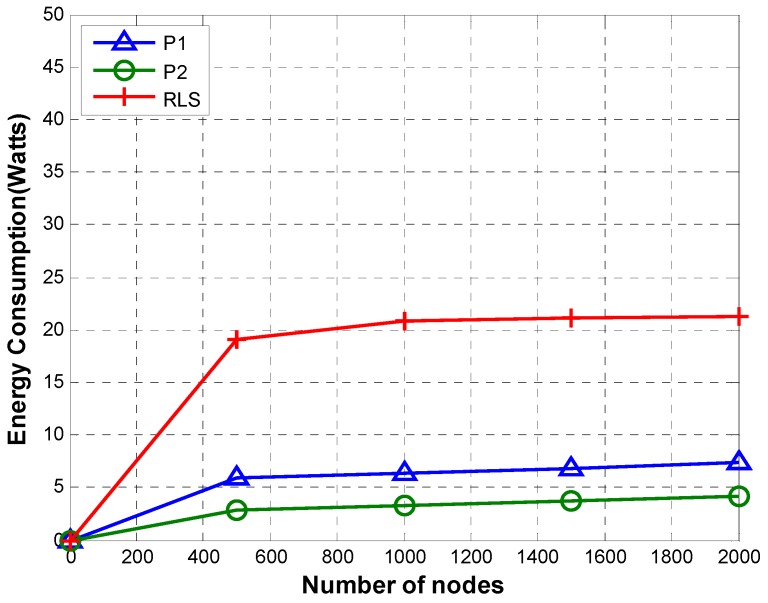
Energy consumption (sparsity).

Figure 16 shows energy consumption of P1, P2, and RLS. Despite a higher communication overhead, the energy consumption of P1 is almost similar to that of P2. This is due to the fact that P1 transmits TRLs with shorter range to reach only its neighbor in the next tier on the path to the reference node. However, in P2, TRLs are sent directly to the reference node with higher transmission power, thus increasing power requirement for transmission of a TRL. As the sparsity increases, the required transmission power also increases, thus increasing energy consumption slightly. In case of RLS, the energy consumption initially increases. This is due to the fact that more nodes are able to reach a bigger number of anchors due to dense network. This results in a bigger number of transmissions by anchor nodes, thus increasing the overall energy consumption. However, as the network gets sparser, fewer sensor nodes can reach fewer anchors, thus reducing the number of transmissions by anchor nodes. This results in smaller energy consumption as the network gets sparser.

#### 5.2.3. Localization Error

**Figure 17:**
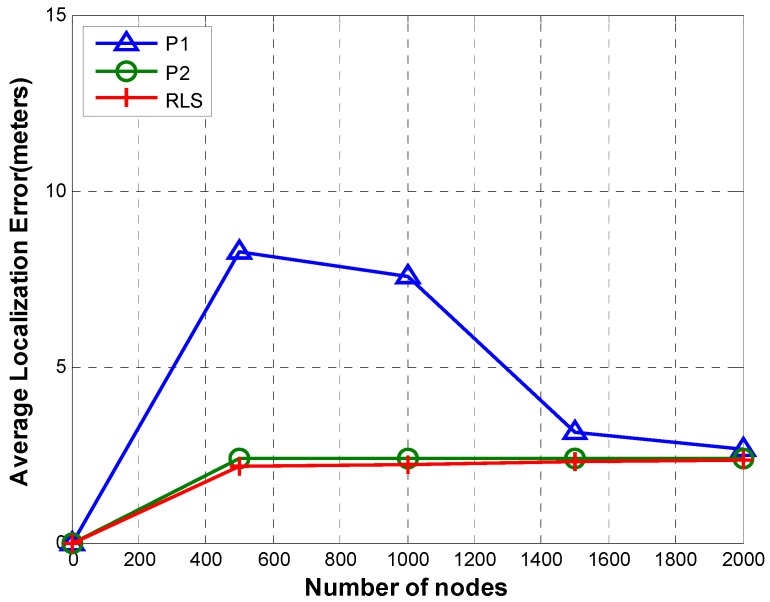
Localization error (sparsity).

Figure 17 shows localization error of P1, P2, and RLS. Localization error in case of P1 and P2 depends on the number of hops taken by TRLs (or ACK in case of tier 1) of node X to reach the reference node.

P2 has almost constant localization error as the sparsity increases from 500 to 2000. In P1 and P2, every node is localized by the reference node using the relative position information in TRLs. In P2, the maximum number of hops from the node being localized (say node X) and the reference node can be 2. The first hop is from node X to the next node (say node Y) in the adjacent tier on the path from node X to the reference node. The second hop is from node Y to the reference node (node Y sends TRL that includes relative position of node X w.r.t. node Y directly to the reference node). The main contribution to the total error is accumulation error. In case of P2, error is always accumulated across two hops irrespective of sparsity. Thus, the difference in errors at different sparsity levels is affected only by other sources of errors (e.g., propagation speed error, depth error). The small variation in error of P1 across different sparsity levels is caused by other sources of errors. For example, the propagation speed error is directly proportional to distance. Therefore, as the sparsity increases, the overall error also increases.

In case of P1, the number of hops from a particular node to the reference node depends on the tier number in which the node is located and the order in which nodes access the channel. For instance, if a node is located in tier X, the distance of the node from the reference node in terms of hops is X hops. When its relative location information (TRL) is sent back to the reference node, the error is accumulated at every hop, thus increasing the overall localization error. When the network gets sparse, partition handling routine is called more frequently due to bigger distances between nodes. Partition handling routing increases ranges of certain nodes. With increased range tiers expand thus resulting in shrinking the number of hops between reference nodes and sensor nodes. As the number of hops decreases, the accumulated error also reduces, thus decreasing the overall localization error as the networks get sparse.

In case of RLS, the localization error remains low irrespective of sparsity. This is due to the fact that every node can access reference nodes directly. Therefore, error accumulation is avoided, resulting in low localization error.

#### 5.2.4. Localization Coverage

**Figure 18:**
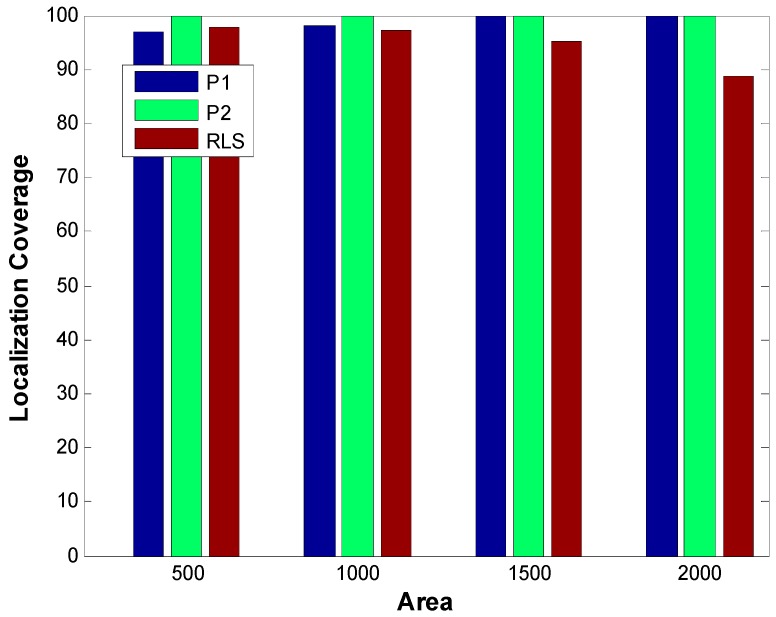
Localization coverage (sparsity).

Figure 18 shows Localization coverage of P1, P2, and RLS. A node is considered unlocalized if it fails to receive a beacon or if its localization error is above a certain threshold value.

Due to low localization error, P2 achieves maximum localization coverage irrespective of sparsity. 2SL has low localization coverage due to two reasons: higher localization error and inability of some nodes to receive a beacon. In P1’s case, higher localization error in denser networks result in slightly lower localization coverage however with better localization error, P1 achieves higher localization coverage. Localization coverage of RLS decreases with increasing sparsity. This is because as sparsity increases, a lower number of nodes can access the required number of anchor nodes.

## 6. Conclusions

This work proposes a two-stage localization scheme for underwater applications that require sensed data to be reported in near real-time. Stage 1 deals with the estimation of relative positions of sensor nodes w.r.t. a reference node whose absolute coordinates are known. For stage 1, two schemes have been proposed; Protocol 1.1 and Protocol 1.2. Protocol 1.1 involves multiple transmissions to fetch TRLs of tier 2 and above to the reference node. Positions of nodes in the received TRL are calculated relative to the reference node based on the positions of the nodes relative to the owner of the TRL and position of the owner of TRL relative to the reference node. In order to further reduce the communication overhead caused by multiple relay transmissions of TRLs in Protocol 1.1, we introduce Protocol 1.2 which improves the performance by sending TRLs directly to the reference node with increased transmission power, thus reducing communication overhead, overall energy consumption, and error accumulation. Avoiding error accumulation results in smaller localization error which in turn improves localization coverage. We also introduce a partition handling mechanism in which only a few selected nodes are directed by the reference node to resolve network partitioning. Performance parameters, which include communication overhead, energy consumption, localization error, and localization coverage, show that the proposed schemes achieve considerable improvement in performance.

## Figures and Tables

**Table 1:** Actions during and at the end of T_WACK_.

Node Type/Ack Status.	One or More ACKs Received	No ACKs Received
Reference node	1. Calculate the relative position of the sender using TDoA. 2. At the end of T_WACK_, compare reference node’s TRL with the list of all nodes. If the two lists match, it means all nodes have been localized relative to the reference node. Therefore, it sends TRL to C&C. However, if the two lists do not match, then it sets T_WTRL_1 (i.e., waiting time to receive TRLs of nodes of tier 1).	It means that none of the nodes is within the default range of the reference node. Therefore, it calls partition handling routine. (explained at the end of Section 3.1)
Any ordinary node x	1. Calculate the relative position of the sender (i.e., the node that sent ACK). 2. At the end of T_WACK_, node x sends its TRL to the upstream node from which it received a beacon. 3. Node x sets T_WTRL_1 (i.e., waiting time for TRL(s) from the next tier down the stream).	No Action

**Table 2:** Actions during and at the end of T_WTRL_.

Node Type/TRL Reception Status	One or More TRLs Received	No TRLs Received
Reference node	1. Apply lemma 2 to calculate relative positions of the newly introduced nodes (i.e., nodes in the received TRL) using: a. The relative position information in received TRL(s) b. The relative position (w.r.t. the reference node) of the node that owns the TRL. 2. At the end of T_WTRL_, the reference node compares its TRL with the list of all nodes. If the two lists match, it means all nodes have been localized relative to the reference node. Therefore, no further action is required. However, if the two lists do not match, then the reference node sets next T_WTRL_ (i.e., waiting time to receive TRLs of nodes of the next tier).	It means that some nodes cannot be accessed with current transmission power. Therefore, the reference node calls partition handling routine which involves increasing the transmission power of certain nodes.
Any ordinary node x	1. At the end of T_WTRL_, node x sends the TRL received from a downstream node to the upstream node from which it received a beacon. 2. Sets next T_WTRL_ i.e., waiting time for TRL’s from tiers further down the stream	No Action

**Table 3:** Setting T_WACK_ and T_WTRL_.

Time/Nodes	Node 1	Node 2	Node 3	Node 4
0	Node 1 transmits beacon Sets T_WACK_ = 2Pd			
Pd	T_WACK_ = Pd	B(beacon) arrives at node 2 Node 2 sets it T_WACK_ = 2Pd Transmits B		
2xPd	2’s B(Ack) reaches node 1 Node 1 calculate the relative position of node 2 All nodes localized = false Sets T_WTRL_1 = 2Pd	Node 2’s T_WACK_ = Pd	Node 2’s B(beacon) arrives at node 3 Node 3 sets it T_WACK_ = 2Pd Transmits B	
3xPd	T_WTRL_1 = Pd	Node 3’s B(Ack) arrives at node 2 T_WACK_ = 0 2 calculates relative position of 3 Sends TRL to 1 2 sets T_WTRL_1 = 2Pd	T_WACK_ = Pd	Node 3’s B(beacon) arrives at node 4 Node 4 sets T_WACK_ = 2Pd Transmits B
4xPd	2’s TRL arrives at 1 1 calculates relative positions of node 3 using Lemma 1 T_WTRL_1 = 0 All nodes localized = false 1 sets T_WTRL_2 = 2Pd	T_WTRL_1 = PD	4’s B (Ack) arrives at 3. 3 calculates relative position of 4 T_WACK_ = 0 3 sends TRL to 2 Sets T_WTRL_1 = 2Pd	T_WACK_ = Pd
5xPd	T_WTRL_2 = Pd	Received TRL from 3 T_WTRL_ = 0 Send received TRL to 1 Set T_WTRL_2 = 2Pd	T_WTRL_1 = Pd	T_WACK_ = 0 Nothing Received No Action
6xPd	T_WTRL_2 = 0 3’s TRL arrives at 1 1 calculates the relative position of 4 using lemma 1 All nodes localized = True Send TRL of node 1 to the surface GPS node	T_WTRL_2 = Pd	T_WTRL_1 = 0 Nothing Received No Action	No Action
7Pd	No Action	T_WTRL_2 = 0 Nothing Received No Action	No Action	No Action

**Table 4:** Simulation parameters.

Parameters	Values
Area	500 × 500, 1000 × 1000, 1500 × 1500, 2000 × 2000
Default Range	250
No. of nodes	20, 40, 60, 80, 100
No. of partitions	1, 2, 3, 4
Packet Size	512 bits
Data rate	5000 bps
Error in propagation speed	0.2 m/s [23]
Error in depth measurement	0.1 m [23]
Mobility based error	0.1 m
Simulation Runs	100

**Table 5:** Evo logic’s S2CR 48/47 modem range vs. energy consumption [41].

Energy Consumption	Range
5.5 W	250 m
8 W	500 m
18 W	1000 m
60 W	Above 1000 m
